# The mitochondrial fission protein Drp1 in liver is required to mitigate NASH and prevents the activation of the mitochondrial ISR

**DOI:** 10.1016/j.molmet.2022.101566

**Published:** 2022-08-06

**Authors:** Janos Steffen, Jennifer Ngo, Sheng-Ping Wang, Kevin Williams, Henning F. Kramer, George Ho, Carlos Rodriguez, Krishna Yekkala, Chidozie Amuzie, Russell Bialecki, Lisa Norquay, Andrea R. Nawrocki, Mark Erion, Alessandro Pocai, Orian S. Shirihai, Marc Liesa

**Affiliations:** 1 Cardiovascular and Metabolic Disease Research, Janssen Research & Development, LLC, 1400 McKean Road, Spring House, PA 19477-0776, USA; 2 Preclinical Safety and Translational Sciences, Janssen Research & Development, LLC, 1400 McKean Road, Spring House, PA 19477-0776, USA; 3 Business Development, Janssen Research & Development, LLC, 1400 McKean Road, Spring House, PA 19477-0776, USA; 4 Department of Chemistry and Biochemistry, UCLA. 607 Charles E. Young Dr., Los Angeles, CA 90095, USA; 5 Department of Medicine, Division of Endocrinology, David Geffen School of Medicine at UCLA. 650 Charles E. Young Dr., Los Angeles, CA 90095, USA; 6 Department of Molecular and Medical Pharmacology, David Geffen School of Medicine at UCLA. 650 Charles E. Young Dr., Los Angeles, CA 90095, USA; 7 Molecular Biology Institute at UCLA, 611 Charles E. Young Drive East, Los Angeles, CA 90095-1570, USA; 8 Institut de Biologia Molecular de Barcelona, IBMB, CSIC, Baldiri Reixac 4-8, Barcelona, Catalonia, 08028, Spain

**Keywords:** NASH, Mitochondria, ISR, Drp1, Oma1, Atf4, ISR, integrated stress response, NASH, non-alcoholic steatohepatitis, NAG, N-acetyl-galactosamine, GAN, Gubra-Amylin-NASH, Drp1, Dynamin related protein 1, NAFLD, non-alcoholic fatty liver disease, NEFA, non-esterified fatty acids

## Abstract

**Objective:**

The mitochondrial fission protein Drp1 was proposed to promote NAFLD, as inhibition of hepatocyte Drp1 early in life prevents liver steatosis induced by high-fat diet in mice. However, whether Drp1-knockdown in older mice can reverse established NASH is unknown.

**Methods:**

N-acetylgalactosamine-siRNA conjugates, an FDA approved method to deliver siRNA selectively to hepatocytes, were used to knockdown hepatocyte-*Drp1* in mice (NAG-Drp1si). NASH was induced in C57BL/6NTac mice by Gubra-Amylin-NASH diet (D09100310, 40% fat, 22% fructose and 2% cholesterol) and treatment with NAG-Drp1si was started at week 24 of diet. Circulating transaminases, liver histology, gene expression of fibrosis and inflammation markers, and hydroxyproline synthesis determined NASH severity. Liver NEFA and triglycerides were quantified by GC/MS. Mitochondrial function was determined by respirometry. Western blots of Oma1, Opa1, p-eIf2α, as well as transcriptional analyses of Atf4-regulated genes determined ISR engagement.

**Results:**

NAG-Drp1si treatment decreased body weight and induced liver inflammation in adult healthy mice. Increased hepatic Gdf15 production was the major contributor to body-weight loss caused by NAG-Drp1si treatment, as Gdf15 receptor deletion (Gfral KO) prevented the decrease in food intake and mitigated weight loss. NAG-Drp1si activated the Atf4-controlled integrated stress response (ISR) to increase hepatic Gdf15 expression. NAG-Drp1si in healthy mice caused ER stress and activated the mitochondrial protease Oma1, which are the ER and mitochondrial triggers that activate the Atf4-controlled ISR. Remarkably, induction of NASH was not sufficient to activate Oma1 in liver. However, NAG-Drp1si treatment was sufficient to activate Oma1 in adult mice with NASH, as well as exacerbating NASH-induced ER stress. Consequently, NAG-Drp1si treatment in mice with NASH led to higher ISR activation, exacerbated inflammation, fibrosis and necrosis.

**Conclusion:**

Drp1 mitigates NASH by decreasing ER stress, preventing Oma1 activation and ISR exacerbation. The elevation in Gdf15 actions induced by NAG-Drp1si might represent an adaptive response decreasing the nutrient load to liver when mitochondria are misfunctional. Our study argues against blocking Drp1 in hepatocytes to combat NASH.

## Highlights

• The FDA-approved NAG-siRNA conjugates were used to knock down Drp1 expression in hepatocytes from older mice.
• Drp1 knock down in control mice results in liver damage, decreasing food intake via Gdf15 actions.
• Drp1 knock down decreases respiration and elevates intrahepatic NEFA, activating Oma1 and the mitochondrial ISR.
• IISR, fibrosis, inflammation and hepatocyte cell death are exacerbated by Drp1 knockdown in mice with established NASH.

## 1 Introduction

No pharmacological agents are available to treat non-alcoholic steatohepatitis (NASH), a disease expected to become the first cause for liver transplantation in Western societies [1]. The presence of lipid accumulation in hepatocytes, together with lobular inflammation and hepatocyte ballooning, is used to diagnose NASH [1]. As lipid accumulation can precede hepatitis and fibrosis, it is thought that changes in hepatocyte lipid metabolism play a major role in initiating inflammation. Accordingly, preclinical studies and clinical trials testing approaches to combat NASH aim to reverse lipid accumulation in hepatocytes. Some of these approaches include the use of molecules that increase mitochondrial fatty acid oxidation in hepatocytes [2,3], that inhibit enzymes catalyzing lipid synthesis in hepatocytes [4] and that limit the availability of dietary fat and carbohydrates to hepatocytes, while concurrently stimulating hepatic catabolism [3].

Mitochondria are key determinants of lipid metabolism in fatty liver disease [5]. In patients with insulin resistance and fatty liver, fat oxidation in mitochondria is upregulated to support the increased demand for both ATP and redox processes that sustain elevated glucose production [6,7]. The upregulation in mitochondrial function in NAFLD not only includes elevated fat oxidation, but also increased TCA fluxes [7], the latter providing carbon intermediates that fuel fatty acid synthesis [8]. Elevated mitochondrial ROS production, caused by this permanent increase in mitochondrial oxidative function in fatty livers, was proposed to initiate hepatitis by causing oxidative damage [9,10]. Consequently, a failure or maladaptation of mitochondria to this increased metabolic load appears to be the key event triggering the transition to NASH. As a result, targeting mitochondria is an appealing strategy to resolve hepatic lipid accumulation, counteract oxidative damage, and potentially treat NASH.

Mitochondrial function and ROS production are strongly determined by the ability of an individual mitochondrion to engage in fusion and fission events and move within the cell, processes collectively known as mitochondrial dynamics [11]. The shape of mitochondria itself not only determines their function in synthesizing ATP and producing ROS, but also their effective elimination by mitophagy [12]. As multiple stresses that primarily increase ROS production, impair mitochondrial ATP synthesis and/or activate cell death cause mitochondrial fragmentation, there was an early interest in blocking Drp1-mediated fragmentation to treat different diseases associated with mitochondrial dysfunction.

In the context of NAFLD, hepatocyte-restricted Drp1 inhibition protected mice from fatty liver induced by high fat diet feeding [13,14]. Constitutive hepatic Drp1 deletion largely prevented body fat gain, glucose intolerance and hepatic triglyceride accumulation [13]. The metabolic benefits induced by Drp1 deletion were attributed to an increase in Fgf21 secretion from the liver to the bloodstream, with secreted Fgf21 activating energy expenditure in other tissues [13]. However, an increase in hepatic ER stress and liver damage explained the increase in hepatic Fgf21 secretion caused by deletion of Drp1 in hepatocytes [13]. On the other hand, decreasing Drp1 activity by expressing a dominant negative form of Drp1 (K38A) prevented steatosis without causing ER stress or liver damage [14]. In this case, the proposed mechanism for protection from steatosis was improved hepatic lipid excretion, combined with elevated fat expenditure induced by mitochondrial uncoupling in hepatocytes [14]. This latter study supported that a Drp1 knockdown approach could hold promise in reversing not just simple steatosis, but NASH as well.

Delivery of siRNAs conjugated to N-acetyl-galactosamine (NAG) is a safe approach to knockdown gene expression specifically in hepatocytes. Accordingly, NAG-siRNA based therapies are approved by the FDA and EMA to treat acute hepatic porphyria, a liver disease with an onset mostly in females ranging from 14 to 45 years old [15,16]. Therefore, NAG mediated delivery of Drp1-siRNA in mice with established NASH is the best preclinical approach to determine whether Drp1 knockdown holds translational promise to reverse NASH in humans. In contrast, the previous preclinical approaches published held less translational relevance: Drp1 was knocked out or its activity decreased prior to high fat diet feeding, using strategies that do not have FDA approval for liver disease. In this study, we define the safety and efficacy of an FDA approved approach to knockdown Drp1 selectively in hepatocytes of mice with established NASH. We define a novel toxic effect of Drp1 knockdown in adult liver, which supports that inhibiting hepatic Drp1 activity using NAG-siRNA conjugates might not be a feasible therapy to treat liver disease.

## 2 Materials and methods

### 2.1 Mice handling and NAG-Drp1-siRNA delivery for the Drp1 knockdown safety study

All procedures described below were approved by the Janssen Institutional Animal Care and Use Committee. All mice received humane care according to the criteria outlined in the ‘‘Guide for the Care and Use of Laboratory Animals’’ published by the National Institutes of Health (NIH publication 86–23 revised 1985). An RNA interference (RNAi) trigger was designed and synthesized by Arrowhead Pharmaceuticals Inc (Pasadena, CA), a company specialized to generate siRNA for clinical trials. The RNAi trigger contains a double-stranded oligonucleotide covalently linked to an N-acetyl-galactosamine (NAG) ligand for hepatocyte targeting, which silenced mouse Drp1 gene expression by targeting a 20-nucleotide region (sequence 5′-UGAGCUAGCGUAUAUCAACA) of *Drp1* mRNA. A similar *in-silico* strategy to identify potential off-target genes in human siRNA was used here for mice. Briefly, the reverse complement of the relevant bases of the antisense strand (AS) sequence of the mouse Drp1 GalNAc-siRNA was aligned with all mouse transcript sequences present in the NCBI database. Mouse genes with alignments having 3 or fewer mismatches to the AS were counted. The results showed that there were no potential off-target genes identified with perfect complementarity or with 1 mismatch to the AS.

Ten weeks old C57BL/6NTac male mice were fed regular chow diet (5053; LabDiet, 5% dietary fat; 3.42 kcal/g) and maintained in a 12/12 h light–dark cycle, with bedding and cage enrichment, free access to food and water in a temperature-controlled environment (22 °C). These mice were treated with a weekly subcutaneous injection of either PBS or NAG-Drp1-siRNA (10 mg/kg, 5 ml/kg) for a total of four injections performed in the light cycle. Body weight was monitored weekly and mice were euthanized via CO_2_ seven days after the 4th dose. The whole liver was weighed using a precision scale and liver fat mass was measured by EchoMRI.

### 2.2 NASH model and NAG-Drp1-siRNA efficacy study

At Taconic Farms, 6-week-old C57BL/6NTac males were fed a GAN diet (Research Diets, D09100310, 40% fat, 22% fructose and 2% cholesterol) or a regular chow diet (5053; LabDiet, 5% dietary fat; 3.42 kcal/g). After 22 weeks of diet (28 weeks of age), GAN and chow diet-fed mice were shipped to Janssen facilities. After 2 weeks of acclimation under the same GAN and regular chow diets respectively, mice were injected with vehicle (PBS) or NAG-Drp1si. Two days before the injection, plasma (EDTA) was collected via tail bleeds for baseline ALT/AST measurements. These ALT/AST and body weight values were used to assign mice to vehicle and NAG-Drp1si groups prior treatment, with these assignments equalizing ALT/AST, body weight values, as well as their variance between groups (n = 12 per GAN group). Eight age-matched chow diet-fed mice injected with PBS served as vehicle controls for the GAN diet. Vehicle injection is the standard control used for NAG-siRNA treatment in humans and thus in preclinical studies aiming to bring NAG-siRNA into the clinic, as it is the best control to determine safety and efficacy [17]. Subcutaneous injections of PBS (5 ml/kg) or Drp1 NAG-siRNA (10 mg/kg, 5 ml/kg) were administered once a week. Twelve-weeks post-treatment and 3 h after fasting, mice were euthanized using CO_2_. Plasma (EDTA) was collected via cardiac puncture. The liver was weighed on a scale, and liver fat mass was measured by EchoMRI.

### 2.3 Liver fixation and histopathology

The right medial and left lateral lobes were trimmed and immersion-fixed in phosphate-buffered 10% formaldehyde for 48 h and then switched to 70% EtOH. The lobes were embedded in blocks of paraffin. All blocks were sectioned into 5 μm paraffin sections and slides were stained with hematoxylin and eosin (H&E) or Picrosirius red (PSR). The pathologist (KY) was initially blinded to the treatment groups. The PSR-stained slides were examined microscopically, and deposition of PSR-stained collagen was diagnosed as fibrosis, with a score provided according to the area stained by PSR. The H&E-stained sections were imaged and blindly analyzed for the presence of necrosis, biliary/oval cell hyperplasia, pigmented macrophages, macrovesicular and microvesicular steatosis, number of inflammatory foci, defined as a cluster of 5 or more cells per 5 fields (100X), and degeneration/hypertrophy [18]. A NAS score for each sample was generated by calculating the sum of macro and microvesicular steatosis, degeneration/hypertrophy, and inflammation as per modified scoring criteria.

### 2.4 Quantitative assessment of immunohistochemistry analyses (IHC)

Paraffin embedded sections were de-paraffinated in xylene and rehydrated in series of graded ethanol. Type I collagen (Southern Biotech, 1310–01), alpha-smooth muscle actin (α-Sma) (Abcam, Ab124964) and Cd11 b (AbCam, 133357) immunostainings were performed using standard procedures. Briefly, after antigen retrieval and blocking of endogenous peroxidase activity, slides were incubated with primary antibody, which was detected using a polymeric HRP-linker antibody conjugate, and visualized using DAB as chromogen. Finally, sections are counterstained in hematoxylin and cover-slipped. The image analysis VIS software (Visiopharm, Denmark) was used to quantify the liver section area stained by DAB.

### 2.5 Respirometry on previously frozen liver samples

Frozen livers were thawed in ice-cold PBS, then minced with scissors, transferred to 2 ml of ice-cold MAS buffer (70 mM sucrose, 220 mM mannitol, 5 mM KH_2_PO_4_, 5 mM MgCl_2_, 1 mM EGTA, 2 mM HEPES pH 7.4) and homogenized in a Teflon-glass Potter-Elvehjem homogenizer, applying 6–9 strokes. Homogenates were then centrifuged at 1,000 g for 10 min at 4 °C and the supernatant was used for the analysis. Protein concentration in the supernatant was determined using Pierce™ BCA Protein Assay Kit (Thermo Fisher). Eight micrograms of protein were loaded per well of a Seahorse XF96 microplate in 20 μl of MAS as published [19]. The loaded plate was centrifuged at 2,000 g for 5 min at 4 °C (stopping the rotor without brakes). Then, an additional 130 μl of MAS buffer containing cytochrome c at 10 μg/ml (final concentration) was added per well. Injections to deliver substrates in each well, during the oxygen consumption rates (OCR) measured in the XF96 Extracellular Analyzer (Agilent), occurred in the following order: 1 mM NADH or 5 mM succinate + 2 μM rotenone were injected first from port A; 2 μM rotenone + 4 μM antimycin A from port B; 0.5 mM TMPD + 1 mM ascorbic acid from port C and 50 mM azide from port D. These are final concentrations, with 10X being loaded in each port.

Wave software (Agilent) was used to calculate OCR. Rates were normalized by protein loaded and by mitochondrial content, the latter determined quantifying MitoTracker Deep Red staining in liver homogenates (see below). Complex I-, II-, and IV-dependent respiration was calculated by the following formulas: Maximal Complex 1 -driven respiration = OCR NADH – OCR antimycin, Maximal Complex 2 -driven respiration = OCR Succinate + Rotenone – OCR antimycin and Maximal Complex 4 activity = OCR TMPD + Ascorbate – OCR azide.

### 2.6 Mitochondrial content quantification in homogenates using MitoTracker Deep Red (MTDR)

MTDR mitochondrial content quantification was performed as published [19]. Briefly, 20 μl of liver homogenate (8 μg) were seeded per well on a clear-bottom black 96-well plate (Corning) in 100 μl of MAS (see 2.5) containing MitoTracker Deep Red (MTDR, Thermo Fisher) at 0.5 μM final concentration. This liver homogenate with MTDR was incubated at 37 °C for 10 min. Plates were centrifuged at 2,000 g for 5 min at 4 °C (no brakes), and supernatant was carefully removed. Finally, 100 μl of MAS was added per well. MTDR was excited at 625 nm and its emission recorded at 670 nm. Mitochondrial content was calculated as MTDR signal, minus background fluorescence (from wells without protein loaded), per milligram of protein. Fluorescence was measured using the Spark 20 M Microplate Reader (Tecan).

### 2.7 Plasma analyte measurements, western blot, qPCR, mass spectrometry analyses of liver lipids and hydroxyproline synthesis in liver

These methods are explained in detail in the Supplementary information.

### 2.8 Mitochondrial imaging in liver sections and morphology analyses

OCT embedded sections were fixed with acetone for 15 min at room temperature and stained with anti-Tomm20 antibody conjugated with a 647 nm excitation fluorophore (Abcam, ab209606). A Zeiss LSM 880 confocal microscope in Airyscan mode was used to visualize mitochondria in liver sections, exciting the sample with a 633 nm laser and imaging the sections with a Zeiss 63 × /1.4NA oil immersion objective. 20 fields of images containing 3–6 cells per liver section were collected for 6 mice per experimental condition. Mitochondria within areas of interest were individualized through the CellProfiler cell image analysis software (https://cellprofiler.org/). To minimize background, images were subjected to a median filter. Segmentation of mitochondria was performed by utilizing a global, two-class, otsu-thresholding method and minimizing the weighted-variance to shape. Identified objects were then subjected to automated shape descriptor analysis, which was used to measure the area of each individual mitochondrion. Data are presented as symbols that represent the average individual mitochondrion area per imaged field. Representative images shown were adjusted in brightness and contrast for better visualization.

### 2.9 Statistical analysis

Comparisons between groups were made by one-way analysis of variance (ANOVA). Correction for multiple comparisons was made by Tukey or Sidak's tests when appropriate. Pairwise comparisons were made by two-sided Student's t-test. Differences were considered statistically significant at P < 0.05. In the figures, asterisks denote statistical significance (∗P < 0.05; ∗∗P < 0.01; ∗∗∗P < 0.001; ∗∗∗∗P < 0.0001). Data were analyzed with GraphPad Prism v7.02. In the figures, each point represents a biological replicate. Error bars represent standard error of the mean or standard deviation, as stated in the legends.

## 3 Results

### 3.1 Hepatocyte-specific Drp1 knockdown (NAG-Drp1si) in healthy mice induces liver damage and increases serum Gdf15 levels, resulting in decreased food intake and body weight

The major goal of our study was to determine whether NAG-Drp1si treatment is safe and efficacious in pre-clinical mouse models of NASH, to make an informed decision on whether NAG-Drp1si could advance to phase I clinical trials. In humans, weekly injections of low NAG-siRNA conjugate concentrations for 4 weeks (total of 4 injections) are safe and highly efficacious decreasing the expression of the gene of interest [17]. This efficacy and safety in humans was established by comparing the effects of NAG-siRNA treatment versus individuals injected with vehicle [17]. The reason for using vehicle as a control in humans is that a scramble siRNA is a drug with its own potential safety issues. Therefore, given that the injection of scramble siRNA will only confirm an absence of the *off-target* effects observed *in silico*, the use of scramble siRNA sequences as control in humans has an unacceptable risk/benefit ratio. The safety of using hepatocyte-targeted siRNA in humans and mice has already been demonstrated, which means that any toxicity or benefit caused by NAG-Drp1si injections, when compared to vehicle treatment in mice, is sufficient to decide whether NAG-Drp1si should advance to clinical trials. Moreover, using mice injected with vehicle as the control group allows to establish a more robust safety and efficacy profile, as the cofounding aspect of potential off-target effects observed with a specific scramble siRNA is avoided.

**Figure 1:**
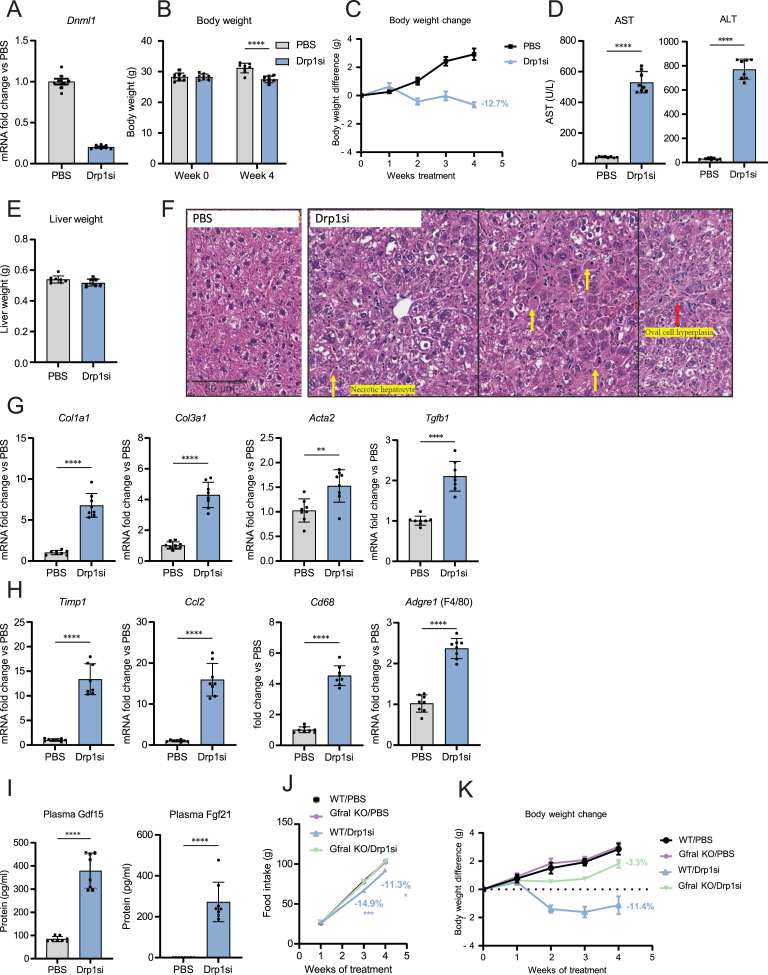
Hepatocyte-specific knockdown of Drp1 (NAG-Drp1si) in control mice induces liver inflammation and fibrosis, as well as body weight loss due to Gdf15-mediated reduction in food intake. **(A**–**I)** Ten-week-old C57BL/6NTac male mice injected weekly with PBS (control vehicle, n = 8) or NAG-Drp1-siRNA (NAG-Drp1si, n = 8) for 4 weeks and their livers analyzed at 14 weeks of age. Data presented are the mean ± SEM or SD and ∗p < 0.05, ∗∗∗p < 0.001, ∗∗∗∗p < 0.0001 one-way ANOVA or two-sided Student's t-test. (**A)** Liver *Dnm1l(Drp1)* mRNA content analyzed by qPCR. (**B)** Total body weight at start (0 weeks) and after treatment with control or NAG-Drp1si. (**C)** Body weight change in grams per week of PBS or NAG-Drp1si treatment. (**D)** Serum ALT and AST levels after PBS or NAG-Drp1si treatments. (**E)** Final liver weight after PBS or NAG-Drp1si treatments. (**F)** Representative Hematoxylin and Eosin (H&E) staining of livers after PBS or NAG-Drp1si treatments. (**G)** Expression of fibrogenesis-related genes analyzed by qPCR in liver after PBS or NAG-Drp1si treatments. (**H)** Expression of Inflammation-related genes by qPCR after PBS or NAG-Drp1si treatments. (**I)** Plasma Fgf21 and Gdf15 protein concentrations measured after PBS or NAG-Drp1si treatments. (**J)** Food intake at each week of PBS or NAG-Drp1si treatments (weekly injections for 4 weeks) of wild type or Gfral knockout (KO) male mice (n = 8). (**K)** Body weight change in grams measured in wild type or Gfral knockout (KO) male mice after control or NAG-Drp1si treatments (n = 8).

**Figure 2:**
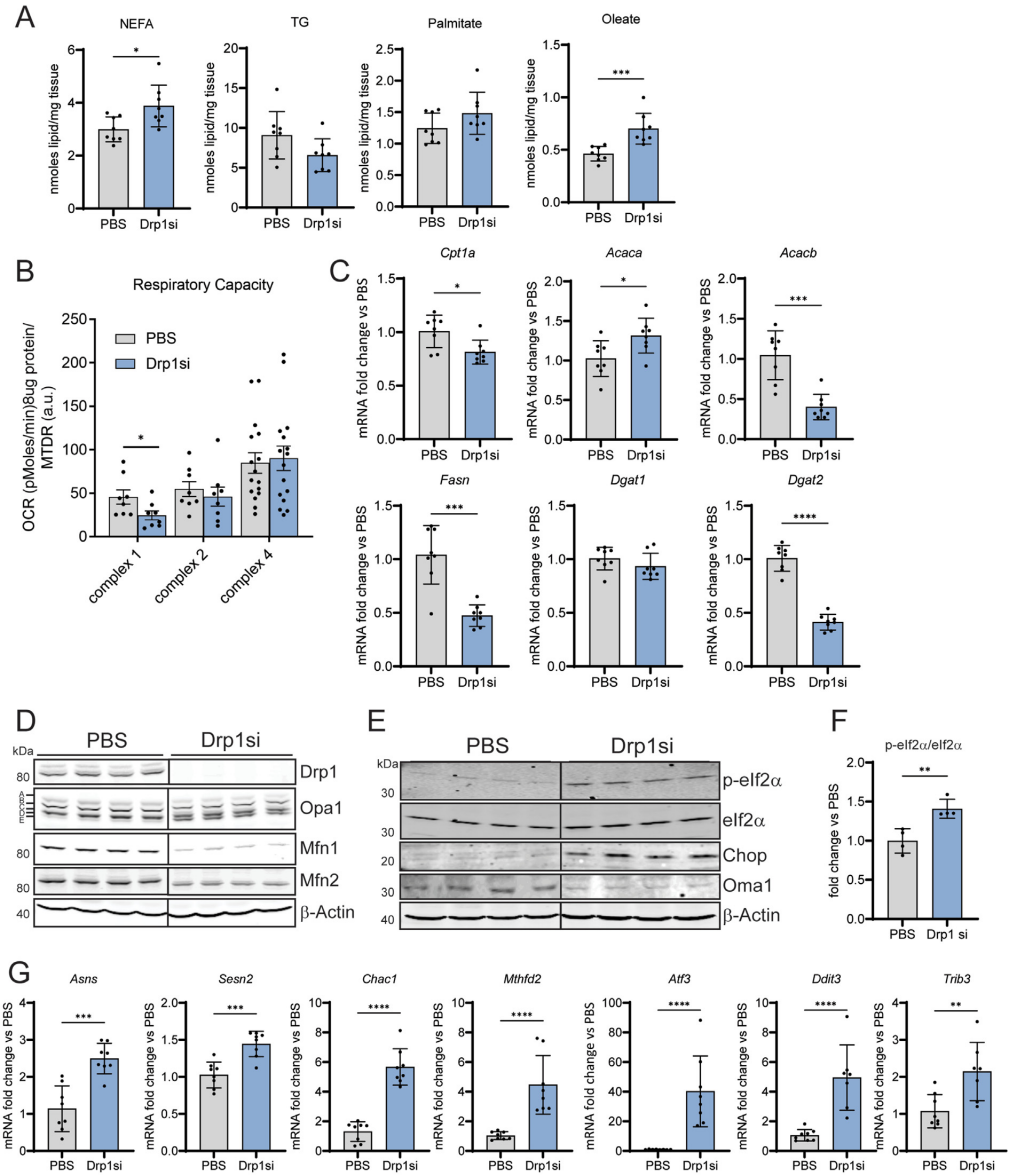
Drp1 knockdown (NAG-Drp1si) in lean mice decreases mitochondrial respiratory capacity, elevates NEFA and activates the mitochondrial integrated stress response (ISR) in liver. (**A-G)** Ten-week-old C57BL/6NTac mice were injected weekly with PBS (control vehicle, n = 8) or NAG Drp1 siRNA (NAG-Drp1si, n = 8) for 4 weeks and their livers analyzed at 14 weeks of age. Data presented are the mean ± SEM or ± SD. ∗p < 0.05, ∗∗p < 0.01, ∗∗∗p < 0.001, ∗∗∗∗p < 0.0001 one-way ANOVA or two-sided Student's t-test. **A)** Total liver content of non-esterified fatty acids (NEFA), palmitate, oleate and triglycerides (TG) were measured by mass spectrometry after PBS or NAG-Drp1si treatments. **(B)** Complex 1 and 2 driven respiration, as well as total complex 4 activity were measured in liver homogenates after control or NAG-Drp1si treatments. Each dot represents the average respiration of each mouse. **(C)** Expression analysis in total liver of genes controlling lipid metabolism and content in liver after PBS or NAG-Drp1si treatments. **(D)** Immunoblot of liver extracts measuring total content of mitochondrial dynamics components Drp1, Mfn1, Mfn2 and Opa1 after PBS or NAG-Drp1si treatments. β-Actin was used as a loading control. The immunoblots had the resolution to detect the 5 Opa1 isoforms (a,b,c,d and e bands) resulting from Opa1 splicing and proteolytic processing. The pattern of Opa1 isoforms in the NAG-Drp1si group is a sign of increased Oma1 activity (decrease a and b, increase in c and e bands). **(E)** Immunoblot of total liver lysates after PBS and NAG-Drp1si treatments to measure the total content of proteins upstream (Oma1, eIf2α, p-eIf2α) and downstream (Chop) of the integrated stress response (ISR) controlled by p-eIf2α-Atf4 axis. Dividing lines separate parts of the same gel that contain different groups of mice. One mouse per lane. **(F)** Densitometric analysis of total eIf2α and the p-eIf2α/eIf2α ratio shown in panel A. Each data point is from one mouse. **(G)** Expression measurements by qPCR of the genes upregulated by p-Eif2α action (*Atf3* and *Ddit3*) and well-known Atf4-controlled genes (*Asns, Sesn2, Chac1*, *Mthfd2* and *Trib3*).

Thus, we first determined the safety and efficacy of using NAG-siRNA conjugates to knockdown hepatocyte Drp1 in healthy mice, using vehicle injected mice as control. The *in-silico* analyses of mouse Drp1 revealed that the siRNA sequence used did not have off-target effects (see Materials and Methods). The NAG-Drp1si conjugates designed were injected weekly in 10-week-old healthy mice for a total of 4 weeks. The 4 injections regimen induced an 80% decrease in *Drp1* mRNA and protein (Figure 1, Figure 2D). Mice stopped gaining weight after the second injection of NAG-Drp1si, resulting in net loss of total body weight (Figure 1B,C) and a 24- and 8-fold increase in circulating ALT and AST respectively after the fourth injection (Figure 1D). No changes in liver weight were observed (Figure 1E) and liver histology revealed clear signs of hepatocyte injury, including presence of oval cell hyperplasia and randomly scattered necrotic hepatocytes (Figure 1F). Furthermore, the expression of fibrogenic (*Col1a1, Col3a1, Acta1 and Tgfb1)* and pro-inflammatory genes (*Timp1, Ccl2, Cd68, Adgre1)* were increased by NAG-Drp1si treatment (Figure 1G,H).

Hepatocytes can regulate body weight by secreting hepatokines that change energy balance. Hepatocyte-specific deletion of Drp1 increased circulating Fgf21, which elevated energy expenditure and appeared to be the main factor explaining weight loss in hepatocyte-Drp1 KO mice [13,14]. We found that treatment with NAG-Drp1si increased both circulating Fgf21 and Gdf15 levels in plasma (Figure 1I). Gdf15 action in the brain was shown to decrease food intake, which was the main action explaining Gdf15-induced weight loss [20]. In agreement with increased circulating Gdf15 levels, NAG-Drp1si treatments reduced food intake (Figure 1J). To determine the exact contribution of Gdf15 on the decrease in food intake and weight loss induced by Drp1 knockdown, we determined the effects of NAG-Drp1si treatment in GDNF family receptor alpha-like knockout mice (Gfral KO), the receptor of Gdf15 [20]. We found that Gfral KO mice largely preserved weight gain after NAG-Drp1si treatment; Gfral KO showed only a 3.3% reduction in body weight when compared to 11.4% reduction in WT mice (Figure 1K). NAG-Drp1si treatment was unable to decrease food intake in Gfral KO mice (Figure 1J), which demonstrated that a reduction in food intake was the greatest contributor to weight loss induced by NAG-Drp1si.

Drp1 knockdown in Gfral KO animals compared to Drp1 knockdown in chow-fed animals yielded no differences in serum liver injury markers (ALT and AST), nutritional stress biomarkers (Fgf21 and Gdf15) and most pro-inflammatory and fibrosis markers (Supplemental Figs. 1A–D). The absence of differences in liver damage between WT and Gfral KO mice supports that the actions of Gdf15 do not contribute to the increase in ALT, AST and Fgf21 induced by NAG-Drp1si treatment. Altogether, these results demonstrate that liver-specific Drp1 knockdown using NAG-siRNA causes hepatic injury in chow diet-fed mice.

### 3.2 Drp1 knockdown in hepatocytes decreases mitochondrial respiratory capacity and induces an accumulation of intrahepatic NEFA, while decreasing total fat accumulation in liver

Steatohepatitis can occur when accumulation of fat inside hepatocytes is large enough to disrupt lipid metabolism, with this disruption initiating inflammation and cell death. To determine whether altered lipid metabolism could have a role initiating liver injury in response to Drp1 knockdown, livers of mice treated with NAG-Drp1si were assessed for total lipid content and composition. Intrahepatic non-esterified fatty acids (NEFA) and triglycerides (TG) were quantified by GC/MS. Drp1 knockdown in lean healthy mice yielded significant increases in total NEFA compared to control, with a trend to decrease total TG content (Figure 2A). The opposite effects on NEFA versus TG suggested that Drp1 knockdown could be inducing a defect in NEFA esterification into TG. When looking at NEFA composition, NAG-Drp1si treated mice showed a highly significant increase in intrahepatic oleate content, but only an increasing trend in palmitate content. The selective increase in oleate supports a defect in fatty acid esterification into TG, as unsaturated fatty acids are the ones preferentially esterified into TG. Therefore, oleate content can be more sensitive to esterification defects. Another possibility is that Drp1 knockdown could be increasing lipolysis and/or NEFA uptake. In any case, our data supports that NEFA-induced lipotoxicity can be a major effector of liver injury caused by Drp1 knockdown.

Drp1 is required for fission and the elimination of damaged mitochondria by mitophagy. Thus, Drp1 activity preserves the quality of mitochondrial oxidative function [21]. Moreover, liver mitochondria show high rates of fatty acid oxidation, which means that their respiratory capacity can determine intrahepatic NEFA content [5]. Consequently, we measured mitochondrial respiratory capacity in liver homogenates from NAG-Drp1si treated mice, as well as the actions of reactive oxygen species (ROS) in their mitochondria. To gain more mechanistic insight on the effects of Drp1 knockdown on mitochondrial oxidative function, we measured respiration driven by individual electron transport chain (ETC) complexes. More specifically, we measured complex 1 and complex 2 driven respiration, as well as total complex 4 activity. To determine the actions of ROS specifically in mitochondria, we measured peroxiredoxin 3 (Prdx3) dimer to monomer ratios, as increased mitochondrial ROS levels directly cause Prdx3 dimerization. The reason is that Prdx3 is a mitochondrial antioxidant enzyme that forms dimers after detoxifying ROS, by generating disulfide bridges between monomers. Thus, a decrease in total Prdx3 content, as well as an increase in Prdx3 dimerization, indicate an increase in mitochondrial ROS production and actions.

First, we observed a significant decrease in complex 1 and a decreasing trend in complex 2 driven respiration in NAG-Drp1si mice after correction per mitochondrial content with MTDR [19], while no changes were observed in total complex 4 function (Figure 2B). These data were showing selective changes in complex 1 activity per mitochondria, with the decrease in complex 1 function being larger than in complex 2 (Figure 2B). Such a differential effect between complexes could be explained by the published effect of mitochondrial ROS causing the degradation of complex 1 degradation, but not of complex 2 [22]. It was also previously published that Drp1 inhibition, by decreasing mitophagy, caused the accumulation of mitochondria that had suffered ROS-mediated damage [21].

To determine whether NAG-Drp1si treatment increased ROS-mediated damage in liver mitochondria, we measured peroxiredoxin 3 (Prdx3) total content and dimerization in liver homogenates. We found that NAG-Drp1si treatment increased Prdx3 dimerization and decreased total Prdx3 protein content, the latter measured in reducing conditions to break the ROS-dependent dimers formed (Supplementary Fig. 2 A, B). After integrating all these data, we interpreted that the decrease in mitochondrial oxidative function induced by Drp1 knockdown could be a consequence of the impairment in the removal of mitochondria damaged by ROS.

Additionally, we observed transcriptional changes induced by NAG-Drp1si treatment that could decrease mitochondrial fat oxidation independently of the changes in complex 1 and 2 activities. We found that NAG-Drp1si decreased the transcript levels of genes regulating fatty acid oxidation (*Cpt1a* and *Acacb*), fatty acid synthesis (*Fasn*), and triglyceride synthesis (*Dgat2*) (Figure 2C). Increased *Acaca* expression suggested that an increase in malonyl-CoA synthesis could be blocking Cpt1 activity as well, which meant that NAG-Drp1si could also be blocking fatty acid entry into mitochondria. Furthermore, the decrease in *Dgat2* expression, an enzyme responsible for esterifying NEFA into TG, could also contribute to the increase in intrahepatic NEFA accumulation observed in NAG-Drp1si livers. In all, our data show NAG-Drp1si hepatocytes limit the transcription of genes executing lipogenesis and mitochondrial fatty acid oxidation, two processes highly dependent on mitochondrial oxidative function [5].

### 3.3 Drp1 knockdown in lean mice increases Oma1 activity and induces the mitochondrial integrated stress response (ISR)

Most forms of mitochondrial stress depolarize and fragment mitochondria, with fragmentation facilitating the removal of damaged mitochondria by mitophagy [21]. The absence of Drp1-mediated fission prevented the separation of damaged mitochondria from the mitochondrial network, a process that is required to eliminate damaged mitochondria by mitophagy [21]. An early molecular process that separates depolarized mitochondria from the mitochondrial network is the activation of Oma1. Oma1 is a metalloprotease that degrades the mitochondrial fusion protein Opa1 and, as a result, blocks the ability of a depolarized mitochondrion to fuse with others [21,23]. Oma1 is located in the inner mitochondrial membrane, where it cleaves the long isoforms of Opa1 (L-Opa1, a and b bands in a blot) that contain the S1 cleavage site into 2 shorter isoforms (s-Opa1, c and e) [23]. Immunoblotting of total liver extracts from NAG-Drp1si mice revealed a decrease in the L-Opa1 isoforms and the corresponding increase in Oma1-generated Opa1 cleavage products, the S-Opa1 isoforms (bands c and e) (Figure 2D). To further confirm that Oma1 was activated by Drp1 knockdown, we measured the effects of NAG-Drp1si treatment on total Oma1 protein content. The reason is that Oma1 activation causes its autocatalytic cleavage, effectively decreasing total Oma1 content itself [23]. Accordingly, we observed that NAG-Drp1si decreased total Oma1 content (Figure 2E), without changing Oma1 mRNA levels (Supplementary Fig. 2C).

We also observed a decrease in the protein content of Mitofusins, Mfn1 and Mfn2, which elongate mitochondria by executing outer membrane fusion (Figure 2E). This decrease appeared to be caused by a post-transcriptional event, as Mfn1/2 mRNA content was not changed by Drp1 knockdown (Supplementary Fig. 2C). The reduction in Mitofusin proteins together with L-Opa1 degradation, strongly support the decrease in mitochondrial fusion proteins could be a compensatory response to a blockage in Drp1-mediated fragmentation.

Recent studies demonstrated that Oma1 activity has an additional role beyond cleaving Opa1 to decrease fusion and cause mitochondrial fragmentation. More specifically, Oma1-mediated cleavage generates peptides that activate the integrated stress response (ISR), which is executed by the transcription factor Atf4 [24,25]. Mechanistically, Oma1-derived peptides directly activate Hri kinase, which phosphorylates eIf2α to block general translation and selectively increase Atf4 protein content [24,25]. Showing an engagement of the mitochondrial ISR induced by Drp1 knockdown, we observed a marked increase in eIf2α phosphorylation and total eIf2α content in livers from NAG-Drp1si treated mice (Figure 2E,F). Moreover, we observed an elevation in Chop/Ddit3 protein, whose expression is controlled by Atf4 (Figure 2E). To further confirm the increases in phosphorylated eIf2α and Atf4 activities, we measured multiple transcripts downstream of p-eIf2α (*Atf3* and *Ddit3*) [26] and other well-known Atf4-regulated genes (*Asns, Sesn2, Chac1*, *Mthfd2* and *Trib3*) [27]. All these transcripts were upregulated in livers from mice treated with NAG-Drp1si (Figure 2G), confirming the activation of the mitochondrial ISR induced by Drp1 downregulation in liver.

### 3.4 Drp1 knockdown in mice mitigates liver steatosis, circulating hyperlipidemia, body weight gain and the increase in circulating ALT induced by NASH

**Figure 3:**
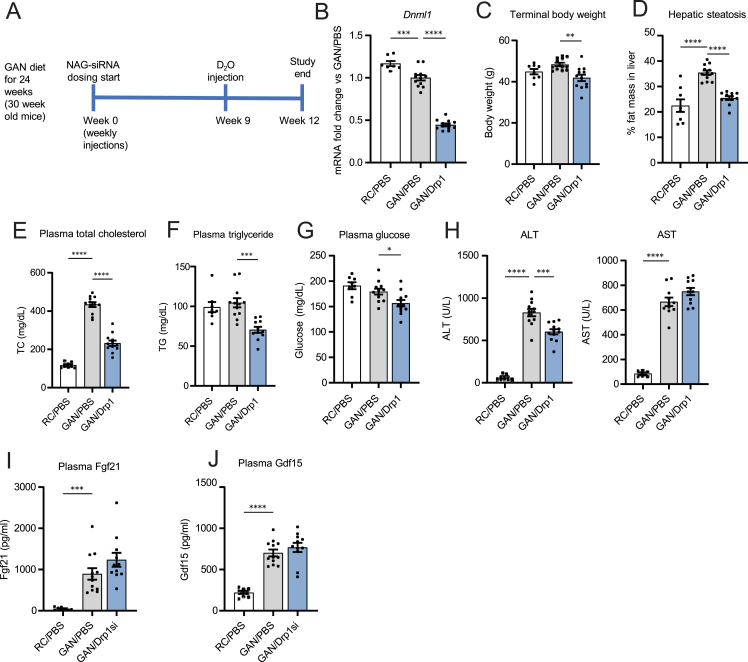
Drp1 knockdown mitigates liver steatosis, circulating hyperlipidemia, body weight gain and the increase in circulating ALT, but not of AST, in mice with GAN diet induced NASH. **(A)** Study design for testing hepatocyte-specific Drp1 knockdown efficacy in the preclinical NASH mouse model of GAN diet feeding for a total of 36 weeks. **(B-J)** Mice fed regular chow (RC) or GAN diet for 24 weeks before being injected weekly with PBS (control vehicle) or NAG Drp1 siRNA (NAG-Drp1si) for 12 weeks. Data presented are the mean ± SEM. RC/PBS n = 8, GAN/PBS n = 12, GAN/NAG-Drp1si n = 12, ∗p < 0.05, ∗∗p < 0.01, ∗∗∗p < 0.001, ∗∗∗∗p < 0.0001 one-way ANOVA. **B)** Liver *Dnm1l* mRNA content analyzed by qPCR. **(C)** Terminal body weights. **(D)** Percent of liver fat mass. **(E)** Plasma cholesterol (TC), **(F)** triglycerides (TG) and **(G)** fed glycemia. **(H)** Circulating ALT and AST levels. **(I)** Plasma Fgf21 protein content. **J)** Plasma Gdf15 protein content.

Liver injury and activation of the ISR induced by NAG-Drp1si treatment in healthy mice argues against using siRNA to knockdown Drp1 as a therapeutic approach to mitigate liver damage. However, it was still possible that Drp1 downregulation could induce benefits exclusively in mice with NASH. This possibility was justified by the expectation that NASH could be hyperactivating Drp1 function, as mouse models of simple steatosis showed increased Drp1 expression [13,14]. To test this possibility, Drp1 was knocked-down in mice with NASH, by injecting NAG-Drp1-siRNA to mice fed for 24 weeks with Gubra-Amylin-NASH (GAN) diet (Figure 3A). A total of 12 weekly injections were provided, which meant that the experiment was finalized at 36 weeks of GAN diet feeding (Figure 3A). Livers from mice fed a GAN diet have a higher translational relevance as a model of NASH, because they recapitulate better the histopathological, transcriptional, and metabolic characteristics observed in human NASH, as opposed to traditional high fat-diet feeding [28].

Remarkably, GAN diet feeding induced a downregulation in Drp1 expression (Figure 3B). As expected, NAG-Drp1si treatments further decreased Drp1 mRNA content in GAN-diet fed mice (Figure 3B). This result was already challenging the conclusion that an increase in Drp1 transcription observed in mice with simple steatosis [14] could contribute to the transition to NASH. Thus, it is a possibility that decreased Drp1 function is an event facilitating the transition from simple steatosis to NASH.

Drp1 downregulation in GAN diet-fed mice decreased their body weight, liver fat mass, fasting plasma cholesterol, plasma triglycerides and glycemia (Figure 3C–G), as well as lowering insulinemia and HOMA IR values (Supplementary Figure 3 A,B). Despite these reductions in plasma lipids and glycemia (Figure 3E–G), AST levels remained high in NAG-Drp1si mice with NASH but were not higher than control mice with NASH (Figure 3H). Indeed, even a decrease in ALT was observed in NAG-Drp1si treated mice with NASH (Figure 3H). A decrease in circulating ALT, in liver triglycerides, as well as in hyperglycemia and body weight, can also occur in cirrhosis, when functional hepatocytes are replaced by fibrotic tissue. Another option was that Drp1 knockdown in liver was inducing both hepatic and metabolic benefits in GAN-diet fed mice.

Supporting the latter conclusion, the NASH-induced increases in circulating Fgf21 and Gdf15 were not further increased by downregulation of Drp1 (Figure 3I,J). Thus, the weight-loss induced by Drp1 knockdown in mice with NASH cannot be attributed to neither an increase in Fgf21 nor Gdf15 levels, as we observed in chow diet fed mice. These latter results also suggested that NASH induced-ER stress could already be maxing out the increase in hepatic Fgf21 and Gdf15 expression. Alternatively, GAN-diet can induce ER stress in other organs, such as muscle and adipose tissue. A greater contribution of other organs to circulating Gdf15 and Fgf21 could also explain why Drp1si in livers with NASH cannot further increase circulating Gdf15 and Fgf21. Thus, with this partial phenotyping, one could draw opposing conclusions: Drp1 knockdown could be metabolically beneficial or detrimental in mice with NASH.

### 3.5 Drp1 knockdown in mice with NASH decreases mitochondrial oxidative function and activates the mitochondrial ISR, exacerbating the increase in intrahepatic NEFA induced by NASH

**Figure 4:**
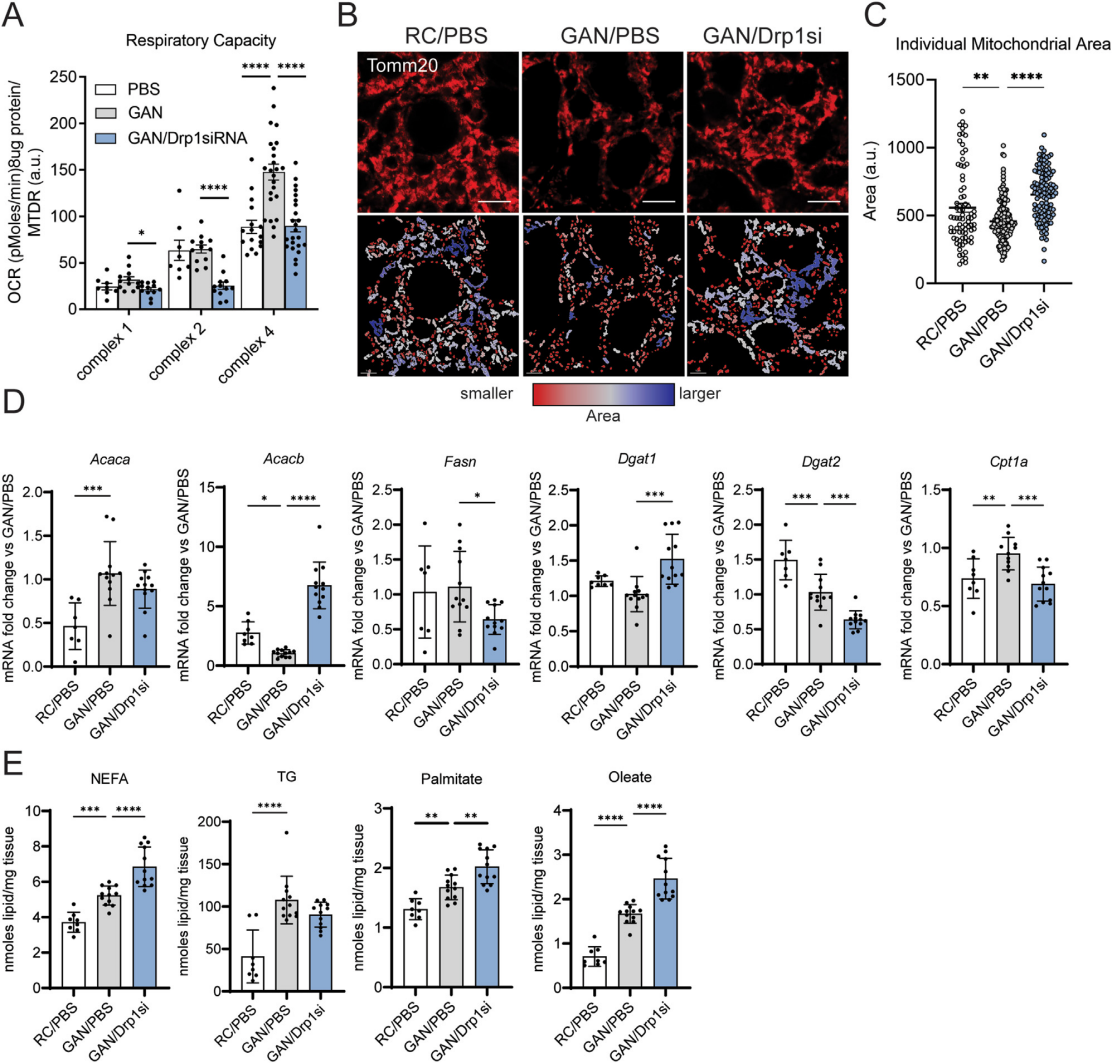
Drp1 knockdown (NAG-Drp1si) in mice with NASH increases mitochondrial size and decreases mitochondrial oxidative function. (A-E) Mice fed regular chow (RC) or GAN diet to induce NASH for 24 weeks before being injected weekly with PBS (control vehicle) or NAG Drp1 siRNA (NAG-Drp1si) for 12 weeks. Data presented are the mean ± SD or ± SEM. RC/PBS n = 8, GAN/PBS n = 12, GAN/NAG-Drp1si n = 12, ∗p < 0.05, ∗∗p < 0.01, ∗∗∗p < 0.001, ∗∗∗∗p < 0.0001 one-way ANOVA. (A) Complex 1 and 2 driven respiration, as well as total complex 4 activity were measured in liver homogenates. Each dot represents the average respiration of each mouse. (B) Mitochondria of liver cryosections stained with anti-Tomm20 antibodies from RC/PBS, GAN/PBS, and GAN/NAG-Drp1si mouse models (Top row). Mitochondria are color coordinated based on small (red), intermediate (white), and large (blue) area (bottom row). (C) Quantification of individual mitochondrion area. Each individual data point shown in the graph is the value of the area averaged for all individual mitochondria segmented in one field, as shown in panel A. A total of 20 fields were imaged for n = 6 mice/group and averaged to calculate SEM. (D) Expression analysis in total liver of genes controlling lipid metabolism and content in liver. (E) Total liver content of non-esterified fatty acids (NEFA), palmitate, oleate and triglycerides (TG) were measured by mass spectrometry.

To determine whether protection from hepatic steatosis induced by Drp1 knockdown in mice with NASH was explained by improved mitochondrial function, we measured mitochondrial oxidative capacity in liver homogenates. In addition, to define the actions of GAN diet feeding and Drp1 knockdown on mitochondrial morphology, we visualized mitochondria in hepatocytes by immunostaining of liver sections. Confirming previous studies demonstrating that mitochondrial oxidative capacity in NAFLD is increased to support lipogenesis and handle the elevation in intrahepatic fat load [9], we found that mice with NASH showed a increasing trend of complex 1 driven respiration and significantly higher complex 4 activity, when compared to chow-fed mice (Figure 4A). This increase in respiratory capacity was associated with mitochondrial fragmentation, as shown by the decrease in the area of individual mitochondrial structures induced by GAN diet feeding (Figure 4B,C).

Remarkably, we found that NAG-Drp1si treatment induced a dramatic decrease in complex 2 activity in mice with NASH: 60% when compared to healthy mice and control mice with NASH (Figure 4A). Moreover, we found that both complex 1 and complex 4 respiration were markedly decreased by NAG-Drp1si treatment (35% and 40% respectively), when compared to mice with NASH treated with vehicle. Such a decrease in oxidative capacity induced by NAG-Drp1si treatment was associated with an increase in the area of individual mitochondria (Figure 4B,C). In this regard, Drp1si mitochondria from GAN-fed mice even showed a trend to be larger than mitochondria from chow diet fed mice (Figure 4B,C). These results argue against Drp1 knockdown preventing liver steatosis by increasing mitochondrial oxidative capacity and confirm that increased fragmentation can enhance mitochondrial oxidative function in liver.

Further supporting that NAG-Drp1si treatment decreased the capacity of mitochondria to oxidize fatty acids, we found that *Cpt1a* expression was reduced and that *Acacb* (ACC2) expression was increased in GAN-diet fed mice treated with NAG-Drp1si (Figure 4D). Moreover, the transcriptional program of lipogenesis was downregulated by NAG-Drp1si treatment in mice with NASH, as shown by the decrease in *Dgat2* and *Fasn* mRNA levels (Figure 4D). Of note, *Dgat1* expression was increased by Drp1 knockdown, suggesting the opposite behavior when compared to other lipogenic genes (Figure 4D). To clarify the effects of NAG-Drp1si treatment on lipid metabolism in mice with NASH, we performed lipidomics of their livers.

As expected, we found that GAN-diet induced NASH caused an increase in intrahepatic NEFA and TGs (Figure 4E). However, while NAG-Drp1si treatment exacerbated the increase in NEFA induced by NASH (both in palmitate and oleate content), this same treatment only induced a non-statistically significant trend to decrease TG content (Figure 4E). Therefore, the effects of NAG-Drp1si selectively increasing intrahepatic NEFAs, while decreasing total intrahepatic fat content, were conserved both in healthy mice and mice with NASH.

**Figure 5:**
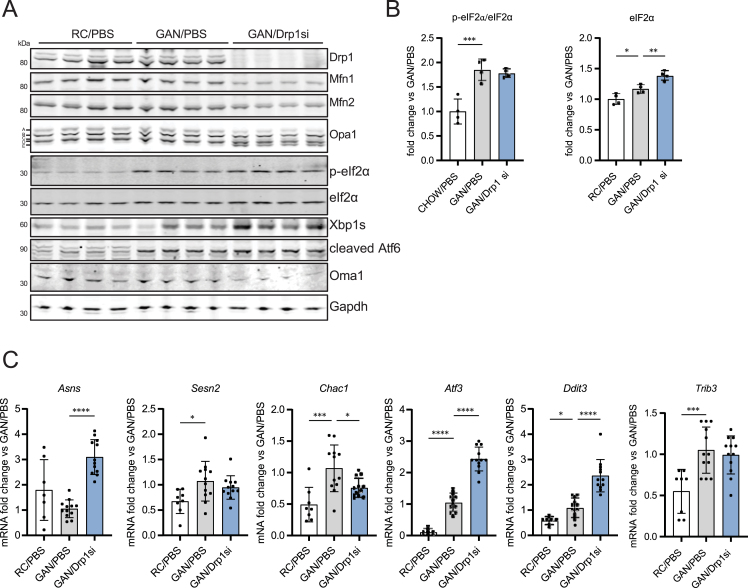
Drp1 knockdown activates the mitochondrial ISR, exacerbating the increase in intrahepatic NEFA induced by NASH. **(A-C)** Mice fed regular chow (RC) or GAN diet for 24 weeks before being injected weekly with PBS (control vehicle) or NAG Drp1 siRNA (NAG-Drp1si) for 12 weeks. Data presented are the mean ± SD. RC/PBS n = 8, GAN/PBS n = 12, GAN/NAG-Drp1si n = 12, ∗p < 0.05, ∗∗p < 0.01, ∗∗∗p < 0.001, ∗∗∗∗p < 0.0001 one-way ANOVA. (A) Immunoblot analyses of liver extracts measuring total content of the mitochondrial dynamics components Drp1, Mfn1, Mfn2 and Opa1 (including Opa1 5 isoforms a-e), as well as of the key markers of mitochondrial ISR activation and ER stress: p-eIf2α, eIf2α, Xbp1s, Atf6 and Oma1. Gapdh was used as a loading control. One mouse per lane. (B) Densitometric analysis of total eIf2α and the p-eIf2α/eIf2α ratio shown in panel A. Each data point is from one mouse. (C) Expression analyses of genes induced by the integrated stress response (ISR) and ER stress measured by qPCR in liver.

A decrease in mitochondrial function and an elevation of intracellular NEFA are sufficient to activate the ISR. Of note, GAN-diet feeding by itself increased intracellular NEFA (Figure 4E), as expected from increased fat and carbohydrate intake under a GAN diet. However, GAN diet-induced NASH was not decreasing mitochondrial oxidative function (Figure 4A). Accordingly, while GAN diet-induced NASH increased p-eIf2α and total eif2α expression (Figure 5A,B), GAN diet-induced NASH by itself did not activate Oma1 nor Opa1 processing (Figure 5A) and was also unable to upregulate *Asns* expression (Figure 5C). These results support that NASH by itself does not engage the mitochondrial arm of the ISR, as *Asns* is a hallmark gene activated by the mitochondrial ISR and a direct target of Atf4. As expected from NASH-induced ER stress and the concomitant increase in p-eif2α and total eif2α expression (Figure 5A,B), other genes whose expression is increased by ER-stress and p-eIf2α activity (*Sesn2*, *Chac1*, *Atf3*, *Ddit3* and *Trib3*) were increased by GAN diet feeding (Figure 5C).

In this context, Drp1 knockdown exacerbated the NASH-induced increase in the expression of total eIf2α, p-eIf2α (Figure 5A,B) and its downstream targets *Atf3 and Ddit3* (Figure 5C). Moreover, Drp1 knockdown markedly increased the expression of the mitochondrial ISR and Atf4-target gene *Asns* (Figure 5C)*.* Of note, Drp1 knockdown in mice with NASH induced a larger increase in total eIf2α content, when compared to the increase induced by NASH itself (Figure 5B). The higher fold increase in total eIf2α content caused that the ratio p-eIf2α/total eIf2α was not higher in NAG-Drp1si treated mice (Figure 5B), despite that total p-eIf2α content and the increase in the expression of ISR target genes were still higher (Figure 5B,C). These results suggested that GAN diet-feeding by itself was causing a maximal increase in the p-eIf2α/eIf2α ratio. Further supporting that GAN diet-feeding alone maximally increased the p-eIf2α/eIf2α ratio, we found that Drp1 knockdown increased the expression of the subunits of Protein Phosphatase 1, Ppp1ca and Ppp1cb. This phosphatase is downstream of Atf4 and is responsible for dephosphorylating p-eIf2α (Supplementary Fig. 3D). Altogether, our data shows that Drp1 knockdown in mice with NASH still activates the mitochondrial branch of the ISR, overriding and/or complementing the ISR that was already initiated by NASH-induced ER stress.

### 3.6 Drp1 knockdown exacerbates hepatic fibrosis and inflammation, inducing hepatocyte death in mice with NASH

**Figure 6:**
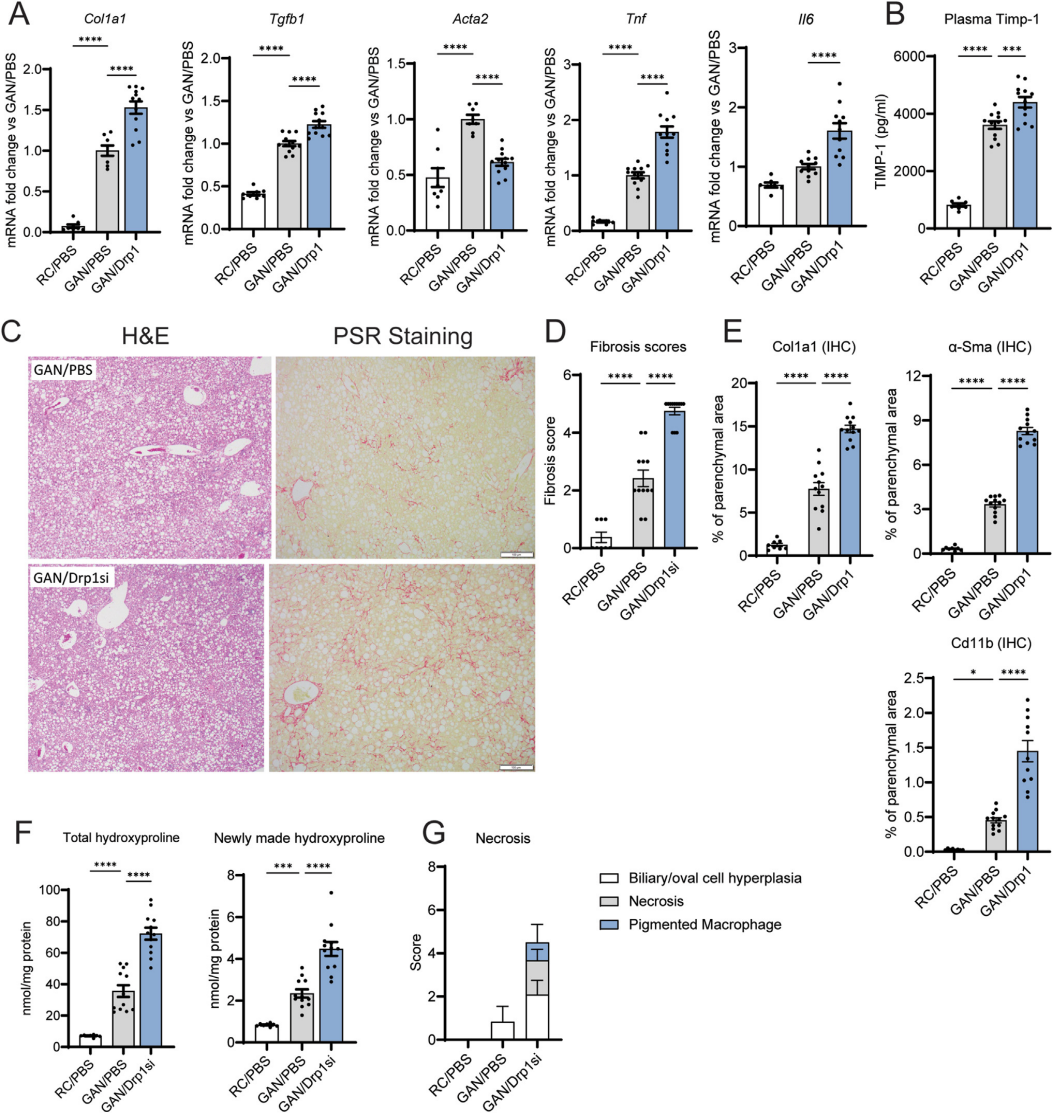
Hepatocyte-specific Drp1 knockdown (NAG-Drp1si) exacerbates fibrosis and inflammation, inducing hepatocyte necrosis in mice with NASH. (A-G) Mice fed regular chow (RC) or GAN diet to induce NASH for 24 weeks before being injected weekly with PBS (control vehicle) or NAG Drp1 siRNA (NAG-Drp1si) for 12 weeks. At week 9, D_2_O was injected to measure the production of hydroxyproline, an indicator for ongoing fibrosis. Data presented are the mean ± SD or mean ± SEM. RC/PBS n = 8, GAN/PBS n = 12, GAN/NAG-Drp1si n = 12, ∗p < 0.05, ∗∗p < 0.01, ∗∗∗p < 0.001, ∗∗∗∗p < 0.0001 one-way ANOVA. A) Gene expression of *Col1a1*, *Tgfb*, *Acta2*, *Tnf*, and *Il6* in liver. B) Plasma Timp-1 protein levels. (C) Representative images of Hematoxylin and Eosin (H&E) and Picrosirius Red (PSR) staining to detect necrosis and fibrosis in liver. (D) Fibrosis scores obtained from Picrosirius Red staining in liver sections (representative in panel C). (E) Quantification of liver areas stained by immunohistochemistry (IHC) using antibodies recognizing Col1a1, α-Sma and Cd11b. (F) Mass spectrometry quantification of total liver hydroxyproline and newly made hydroxyproline labeled with deuterium, with labelling achieved by injecting mice with D_2_O at week 9 of NAG-Drp1si treatments. **(G)** Quantification of the presence of necrosis observed in Hematoxylin and Eosin (H&E) stained sections from liver.

The execution of the ISR by Atf4 in response to mitochondrial injury is a pro-survival response, allowing a cell to carry on vital functions despite mitochondrial dysfunction [24,25]. However, hyperactivation of the ISR and Atf4 can induce cell death [29]. To determine whether the increase in the mitochondrial ISR induced by Drp1 knockdown was adaptive or was exacerbating liver damage, we determined the effects of Drp1 knockdown on liver inflammation, fibrosis, and cell death in GAN diet-fed mice. The expression of inflammation (*Tgf-*β*, Tnf-*α*,* and *Il-6*) and fibrosis markers (*Col1a1*) were significantly upregulated by NAG-Drp1si treatment in mice with GAN-diet induced NASH (Figure 6A). Increased inflammation and fibrosis were confirmed by augmented plasma Timp-1 content (Figure 6B) and in immunohistology analyses (Figure 6C–E). Drp1si-induced exacerbation of fibrosis and inflammation were established by the increase in areas stained with Picrosirius Red (Figure 6D,E) and in areas of parenchyma stained by Col1a1, α-SMA*,* and Cd11b antibodies in liver sections (Figure 6E).

Hydroxyproline production is significantly correlated to hepatic stellate cell activation during liver fibrosis, which makes hydroxyproline a functional biomarker for the progression of liver fibrosis. As such, hydroxyproline synthesis is an independent measure of liver fibrosis. NAG-Drp1si treatment caused an exacerbation in NASH-induced increases to total hydroxyproline content and newly made hydroxyproline (Figure 6F), further confirming that Drp1 knockdown exacerbated liver fibrosis. Finally, Drp1 knockdown caused hepatocyte necrosis in GAN diet fed mice, while the GAN diet itself could not cause necrosis (Figure 6G). Therefore, the stimulation of the ISR by NAG-Drp1si treatment, on top of the ISR induced by GAN diet feeding, could explain the increase in cell necrosis caused by Drp1 knockdown. Taken together, these data imply that NAG-Drp1si treatment in hepatocytes exacerbates NASH by worsening intrahepatic NEFA accumulation and amplifying the ISR, despite decreasing total intrahepatic fat content.

## 4 Discussion

Drp1-mediated mitochondrial fragmentation observed in NAFLD has been proposed to be a maladaptive process that exacerbates hepatic insulin resistance, steatohepatitis and cell death [5]. Accordingly, both deletion and inhibition of hepatocyte-Drp1 activity in a preventative manner protected mice from high-fat diet induced hepatic steatosis, insulin resistance and even body weight gain [13,14]. Furthermore, the mitochondrial adaptor protein that binds Drp1 in the outer membrane to initiate mitochondrial fragmentation, Mff, was shown to be the effector downstream of ceramides to promote insulin resistance and simple steatosis [30]. In this context, we hypothesized that knocking down Drp1 specifically in hepatocytes, using the FDA approved approach of NAG-siRNA conjugates, could reverse established NASH. However, we found that knocking down Drp1 in adult and healthy mice was deleterious to liver function. Drp1 knockdown elevated circulating AST and ALT, increased the expression of pro-inflammatory and fibrosis markers in liver and caused weight loss. Consequently, mice with hepatic Drp1 knockdown via NAG-siRNA showed unequivocal signs of liver damage. Further supporting that NAG-Drp1si induced liver stress, we found an elevation of two circulating cytokines that are secreted by stressed hepatocytes: Gdf15 and Fgf21.

Gdf15 decreases food intake in mice and humans and alters food choice [20]. Furthermore, Gdf15 is a circulating biomarker of mitochondrial disease, with its degree of expression being correlated to disease severity [[31], [32], [33], [34]]. To mediate its actions in decreasing food intake, Gdf15 needs to bind to Gfral, the Gdf15 plasma membrane receptor. The confinement of Gfral expression mostly to the brain supports that Gdf15-mediated decrease in food intake is the major effector of GDF15 actions decreasing body weight [20]. On the other hand, Fgf21 secreted by hepatocytes can induce weight loss by promoting white adipose tissue beiging, which leads to increased energy expenditure [35]. Weight loss induced by NAG-Drp1si treatment was largely prevented in Gfral KO mice, which strongly argues against the conclusion that Fgf21-induced energy expenditure is a major contributor to body weight loss induced by Drp1 knockdown. In agreement with this conclusion, the decrease in food intake induced by Drp1 knockdown was absent in Gfral KO mice.

Next, we aimed to determine how a decrease in Drp1 function elevated Gdf15 and Fgf21 transcription in the liver. Previous studies demonstrated that liver-specific Drp1 KO mice showed an increase in ER stress, which is widely known to increase Gdf15 and Fgf21 transcription by activating the Atf4 transcription factor [13]. Atf4 activation in response to different stresses is known as the integrated stress response (ISR). Indeed, recent evidence demonstrated that not only ER stress, but also mitochondrial stress, can initiate the ISR [24,25]. Mechanistically, stresses that impair mitochondrial ATP production and/or elevate mitochondrial ROS production converge in activating the mitochondrial protease Oma1 [23]. This mitochondrial protease generates peptides that are released to the cytosol to activate the Hri kinase, resulting in eIf2α phosphorylation and Atf4 translocation to the nucleus [24,25]. Therefore, the ISR can be initiated both by mitochondrial dysfunction and ER stress.

In this regard, we found that Drp1 knockdown was sufficient to increase both Oma1 activity and ER stress. Consequently, both mitochondrial dysfunction and ER stress could converge to activate the ISR in NAG-Drp1si mice and, as a result, elevate Gdf15 and Fgf21 expression. Our previous studies demonstrated that Drp1 inhibition in beta cells decreases the removal of damaged mitochondria by mitophagy, resulting in the accumulation of mitochondria damaged by ROS [21]. Given that mitochondrial ROS can selectively induce complex 1 degradation [22], it is a possibility that impaired mitophagy in NAG-Drp1si treated livers caused the accumulation of damaged mitochondria that had both Oma1 activated and complex 1 degraded by ROS. And these damaged mitochondria, by releasing ROS themselves, could damage other mitochondria. Supporting an impairment in mitophagy, we found a selective decrease in complex 1 activity induced by Drp1 knockdown in total liver homogenates, as well as increased ROS actions in mitochondria.

Thus, one could conclude that decreased complex 1 function and the accumulation of dysfunctional mitochondria damaged by ROS can be responsible for the increase in intrahepatic NEFA induced by Drp1 knockdown. Proper complex 1 function and NADH oxidation is not only needed for fatty acid oxidation to proceed, but also to esterify free fatty acids into triglycerides [36]. Moreover, an excess of NEFA is sufficient to induce ER stress and even further depolarize mitochondria to activate Oma1. Supporting the major contribution of elevated NEFA to the liver damage caused by NAG-Drp1si treatment, some genetic manipulations that specifically promote hepatic steatosis by increasing lipid droplet size and NEFA esterification improved hepatic health and glucose metabolism [37].

Previous studies support that the relationship between Drp1 activity and ISR activation is conserved in different tissues. In the brain, Drp1 ablation activates the ISR and increases Fgf21 expression, but the mechanism responsible for ISR activation was not fully characterized [13,38]. Restelli et al. found that Drp1 deletion altered ER-mitochondria contacts, resulting in a change of the curvature of ER and mitochondrial membranes that activated the ER stress sensor Perk [38]. Heme deprivation and changes to amino acid sensitivity because of Drp1 ablation were proposed to further contribute to Atf4 activation [38]. But the exact mitochondrial mechanism on how Drp1 ablation leads to Perk activation remained unclear. Our study suggests that an accumulation of NEFA could explain Perk activation induced by Drp1 ablation.

As in human clinical trials, we used vehicle injected mice as a control group to determine the effects of NAG-Drp1si treatment. The lack of siRNA control in our study could justify testing alternative approaches different to siRNA to decrease Drp1 activity in liver, as there is the possibility that using siRNA by itself can explain some of the toxicity. However, given that we reproduce some on-target effects previously described in liver-specific Drp1 KO models and that the additional toxic effects observed can be a direct consequence of inducing ER stress and changing mitochondrial function (pathways where Drp1 function lies), we conclude that toxicity and increased inflammation is mostly stemming from on-target effects of the siRNA.

Our data shows that NASH induced by GAN diet feeding is not sufficient to activate Oma1, supporting that NASH itself is not engaging the mitochondrial ISR. GAN diet feeding induced hepatic ER stress and increased Atf3 and Atf6 expression, but it could not increase the expression of *Asns*, a specific Atf4 target characteristic of the mitochondrial ISR. These results suggest that Drp1 knockdown specifically engaged the mitochondrial ISR by activating Oma1 and Atf4. Thus, one can speculate that the absence of Drp1-mediated mitochondrial quality control might activate the ISR mostly by inducing mitochondrial dysfunction. Although the GAN diet by itself induces ER stress and activates the ISR, Drp1 knockdown adds an impairment in mitochondrial quality control and an exacerbation of NEFA accumulation. These additive effects caused by Drp1 knockdown can be the main mechanism explaining how NAG-Drp1si treatment exacerbates ER stress, fibrosis and inflammation.

However, it is still an open question if the activation of the mitochondrial ISR induced by Drp1 knockdown is an ineffective adaptive response or, alternatively, contributes to NASH development. The previously published studies showing that Atf4 deletion protected mice from liver steatosis supports that the activation of the mitochondrial ISR might exacerbate NASH [39]. Moreover, our published data showing that Drp1 is needed for proper metabolic adaptation in brown adipose tissue, together with our new data showing downregulation of Drp1 expression in GAN-diet fed mice, strongly support that Drp1 activity should be restored in NASH to counteract NEFA accumulation and ISR hyperactivation.

In all, our study argues against the use of NAG-Drp1-siRNA as a therapeutic strategy to reverse NASH. Furthermore, our study suggests that partial Drp1 inhibition might only be efficacious preventing simple steatosis but might be deleterious in individuals with established NASH.

## Author contributions

J.S., J.N., S·W., H·F·K., L.N., A.P., O·S·S., M.L. conceived the project and designed experiments. J.S., J.N., S·W., K·W., G.H., C.R., K.Y. performed experiments and analyzed data. C.A., R.B., A.N., M.E. participated in discussion of the results. J.S., J.N., S·W., O·S·S., M.L. wrote this manuscript.

## Data Availability

Data will be made available on request.
