# Supplementary material for: The mitochondrial fission protein Drp1 in liver is required to mitigate NASH and prevents the activation of the mitochondrial ISR
Source: Mol Metab. 2022 Aug 6;64:101566. doi: 10.1016/j.molmet.2022.101566 (PMC9420962; doi:10.1016/j.molmet.2022.101566)
Supplement: Multimedia component 1 [file mmc1.docx]

**SUPPLEMENTARY INFORMATION**

**The mitochondrial fission protein Drp1 in liver is required to mitigate NASH and prevents the activation of the mitochondrial ISR.**

Janos Steffen^1*^, Jennifer Ngo^4,5*^, Sheng-Ping Wang^1*^, Kevin Williams^4^, Fritz Kramer^1^, George Ho^1^, Carlos Rodriguez^1^, Krishna Yekkala^2^, Chidozie Amuzie^2^, Russell Bialecki^2^, Lisa Norquay^3^, Andrea Nawrocki^1^, Mark Erion^1^, Alessandro Pocai^1#^, Orian S. Shirihai^5,6#^, Marc Liesa^5,6,7,8#^.

^1^Cardiovascular and Metabolic Disease Research, Janssen Research & Development, LLC, 1400 McKean Road, Spring House, PA 19477-0776, USA

^2^Preclinical Safety and Translational Sciences, Janssen Research & Development, LLC, 1400 McKean Road, Spring House, PA 19477-0776, USA

^3^Business Development, Janssen Research & Development, LLC, 1400 McKean Road, Spring House, PA 19477-0776, USA

^4^Department of Chemistry and Biochemistry, David Geffen School of Medicine at UCLA. 650 Charles E. Young Dr., Los Angeles, CA 90095 USA

^5^Department of Medicine, Division of Endocrinology, David Geffen School of Medicine at UCLA. 650 Charles E. Young Dr., Los Angeles, CA 90095 USA

^6^Department of Molecular and Medical Pharmacology, David Geffen School of Medicine at UCLA. 650 Charles E. Young Dr., Los Angeles, CA 90095 USA.

^7^Molecular Biology Institute, University of California, Los Angeles, CA, USA at UCLA.

^8^Molecular Biology Institute of Barcelona, IBMB-CSIC, Baldiri Reixac 4-8, Barcelona, Catalonia, 08028, Spain.

*Equal contribution as first authors; ^#^Equal contribution as corresponding authors

**SUPPLEMENTARY MATERIALS AND METHODS**

*Mouse plasma analytes measurements*

VET-AXCEL clinical analyzer (Alfa Wassermann) was used to analyze plasma as per manufacturers instructions. The transaminases ALT (Cat# SA1052) and AST (Alfa Wassermann Cat# SA1053), total cholesterol (TCHOL, Cat# SA1010), triglycerides (TRIG, Cat# SA1023) reagents and tests were calibrated with the GEMCAL calibrator (Cat# S1-33) and quality controls tested with Chemical Controls level 1 and 2 (Cat# C1-4 and C1-5 respectively). All calibrations and reagents QCs were performed on a weekly basis before each test run.

*ELISA measurements in plasma*

Plasma Timp-1 (R&D Systems, MTM100), Gdf15 (R&D Systems, MGD150) and Fgf21 (EMD Millipore Corporation, EZRMFGF21-26K) were measured according to the supplied product’s protocol.

*RNA isolation and q-PCR*

Livers were collected in RNA later. Total RNA was isolated from liver tissue using the TRIZol reagent (Thermo Fisher) and further purified with the RNeasy 96 QIAcube HT Kit and QIAcube HT (Qiagen). The obtained total RNA was then reverse transcribed into cDNA using the SuperScript™ IV VILO™ Master Mix (Thermo Fisher) and PCRs were run on a QuantStudio™ 12K Flex Real-Time PCR System using TaqMan Gene Expression Assays and TaqMan Fast Advanced Master Mix (Thermo Fisher). All mRNA expression levels were normalized to Ppia. The 2−ΔΔCt method was used to calculate relative gene expression levels. Primers can be found in the following table:

| Gene name | Thermo Fisher Catalog number |
| --- | --- |
| *Ppia* | Mm02342430-g1 |
| *Col1a1* | Mm00801666-g1 |
| *Acta2* | Mm00725412-s1 |
| *Timp1* | Mm01341361-m1 |
| *Tgfb1* | Mm00178820-m1 |
| *Il6* | Mm00446190-m1 |
| *Tnf* | Mm00443258-m1 |
| *Adgre1* | Mm00802529-m1 |
| *Ccl2* | Mm00441242-m1 |
| *Dnm1l* | Mm01342903_m1 |
| *Cpt1a* | Mm01231183_m1 |
| *Acaca* | Mm01304257_m1 |
| *Acacb* | Mm01204671_m1 |
| *Fasn* | Mm00662319_m1 |
| *Dgat1* | Mm00515643_m1 |
| *Dgat2* | Mm00499536_m1 |
| *Asns* | Mm00803785_m1 |
| *Sesn2* | Mm00460679_m1 |
| *Chac1* | Mm00509926_m1 |
| *Mthfd2* | Mm00485276_m1 |
| *Atf3* | Mm00476032_m1 |
| *Ddit3* | Mm01135937_g1 |
| *Trib3* | Mm00454879_m1 |
| *Col3a1* | Mm00802331_m1 |
| *Cd68* | Mm03047343_m1 |
| *Ppp1ca* | Mm00453295_g1 |
| *Ppp1cb* | Mm01209998_m1 |
| *Ppp1cc* | Mm00849631_s1 |

*Western blot*

Frozen liver tissue was cut on dry ice into approximately 50-60mg pieces and transferred to chilled Lysing Matrix M, 2 ml tubes (MP Biomedicals). Tissue Extraction Reagent II (Thermo Fisher) supplemented with PhosSTOP™ and cOmplete™, EDTA-free Protease Inhibitor Cocktail (Roche Life Science) was added to a final buffer to tissue ratio of 15 µL buffer/mg tissue. Liver tissue was homogenized using the preset mouse liver program on the FastPrep-24™ 5G system (MP Biomedicals) and then centrifuged for 15 min at 21000 x g, 4°C. The supernatant was transferred to a new 1.5 ml tube and the protein concentration was determined with the Pierce™ BCA Protein Assay Kit (Thermo Fisher). 25µg of total protein were denatured at 70°C for 10 min and separated on Novex™ WedgeWell™ 4 to 20%, Tris-Glycine gels (Thermo Fisher) under reducing or non-reducing conditions. Afterward the proteins were transferred on nitrocellulose iBlot™ 2 Transfer Stacks using the iBlot 2 Dry Blotting System (Thermo Fisher). Membranes were blocked in Intercept® (TBS) Blocking Buffer (Licor) for 60 min and then exposed to the primary antibody overnight at 4°C. After applying the secondary antibodies, the membranes were scanned on an Odyssey® CLx Imaging System (Licor). Densitometric analyses were performed with the Image Studio software (Licor). The antibodies can be found in this table:

| Antigen | Dilution | Vendor | Catalogue number |
| --- | --- | --- | --- |
| Opa1 | 1:3000 | BD Bioscience | 612607 |
| Mfn1 | 1:1000 | Proteintech | 13798-1-AP |
| Mfn2 | 1:1000 | Proteintech | 12186-1-AP |
| Oma1 | 1:1000 | Cell Signaling | 95473 |
| β-Actin | 1:20000 | Proteintech | 66009-1-Ig |
| Gapdh | 1:5000 | Santa Cruz | sc-365062 |
| Chop | 1:1000 | Cell Signaling | 2895S |
| p-eIF2α | 1:1000 | Cell Signaling | 3398S |
| eIF2α | 1:1000 | Abcam | ab5369 |
| Dlp1/Drp1 | 1:1000 | BD Bioscience | 611113 |
| Atf6 | 1:1000 | Proteintech | 24169-1-AP |
| Xbp1s | 1:1000 | Biolegend | 658802 |
| Prdx3 | 1:1000 | Abcam | ab73349 |

*Liver hydroxyproline synthesis measurement*

Hydroxyproline synthesis analysis was performed by Metabolic Solutions, Inc. A piece of liver was snap-frozen in liquid nitrogen and stored at -80°C for collagen synthesis. An aliquot (10 mg) of liver was weighed and re-suspended in a 1N HCl solution containing 2 µg m+6 labeled hydroxyproline (^13^C_5_^15^N-OHP). Samples were hydrolyzed at 100°C for 24 hours. A cleanup column is used to remove HCl. Samples are eluted with ammonium hydroxide and dried with nitrogen gas. Hydroxyproline was derivatized using the method described by March (1) with modifications by Matthews, Persola and Campbell (2). The propyl ester is added by addition of 100 µL 1-propanol/acetyl chloride (5:1 v/v). Samples are incubated at 90°C for 20 min. The propylation reagents are evaporated by nitrogen gas and heat (60-70°C). The heptafluorobutyric ester is formed by the addition of 100 µl 0.1M diethylamine in hexane and 10 µl heptafluorobutyric acid (Sigma Aldrich) with incubation at 60°C for 20 min. All reagents are evaporated with nitrogen gas and heat (60-70°C). The sample is reconstituted with 100 µL ethyl acetate. The isotopic enrichment is determined by gas chromatography–mass spectrometric analysis using methane negative chemical ionization (Agilent 5975 EI/CI MSD with an Agilent 7890 GC). A Phenomenex ZB-1MS capillary column is used to separate the derivative of phenylalanine. Selected ion chromatograms are obtained by monitoring ions m/z 545, 546 and 551 for unlabeled HyPro, M+1 HyPro and ^13^C_5_^15^N-OHP, respectively. Incorporation of 2H into OHP was calculated as the molar fraction of molecules with one excess mass unit above the natural abundance fraction (EM1). Fractional turn over (*f*) was calculated as the ratio of the EM1 value in protein-bound OHP to the maximal value possible at the body water enrichment present, which has been empirically determined to be 1.1 * Body Water Enrichment (i.e., f = EM1 / (1.1*BW). The principle behind this calculation has previously been described by Gardner, J.L., et al. Additionally, OHP content in each sample was determined by comparing the abundance in the m+6 551 m/z channel representing the ^13^C_5_^15^N-OHP internal standard in each sample with that the m+0 545 m/z ion. A set of standards with known OHP/^13^C_5_^15^N-OHP concentration ratios was analyzed alongside the samples. Plasma body water samples were analyzed for deuterium enrichment by cavity ring-down spectroscopy using a Liquid Water Isotope Analyzer with automated injection system, version 2 upgrade (Los Gatos Research). Plasma proteins were removed by adding approximately 5 mg zinc sulfate monohydrate to 25-50 µl plasma in a microcentrifuge tube. Samples were vortexed and spun at 8,000 rpm to precipitate proteins. The plasma protein-free supernatant was injected 8 times and the average of the last three measurements used for data analysis. A standard curve was run before and after samples for calculation of deuterium enrichment as delta per mil (parts per thousand) relative to Vienna Standard Mean Ocean Water (VSMOW). Intra-run precision is less than 2 delta per mil (parts per thousand) and inter-run precision is less than 3.5 delta per mil. Delta per mil is converted to atom percent deuterium by a calculation described previously:

*Body water:* Mice were labeled with D_2_O by bolus and drinking water to achieve a constant exposure of D_2_O during the label period of approximately 2.5%. Plasma samples were analyzed for deuterium enrichment.

*Excess M1 (%EM1):* This quantity represents the degree of isotopic incorporation of deuterium into OHP mediated by collagen synthesis *in vivo*.

*Fractional synthesis of OHP (“f”):* This quantity represents the percentage of collagen that was newly-synthesized during the period of label incorporation.

*OHP concentration:* The absolute amount of OHP found in the 10 mg piece of liver analyzed by Metabolic Solutions was determined in mass terms by introducing a known amount of ^13^C_5_^15^N-OHP prior to further processing. The ratio of m0 (representing OHP) to m+6 (representing the ^13^C_5_^15^N-OHP standard) in samples was converted to the OHP content of the sample analyzed, which was divided by grams used to obtain a hydroxyproline concentration value for the tissue*.* Note that by multiplying fractional synthesis (f) with total OHP concentration, one can determine the total mass of OHP in newly-synthesized collagen.

*Shotgun Lipidomics Analysis*

Frozen tissues (50-100 mg) were transferred to extraction tubes with PBS. A modified Bligh and Dyer extraction is carried out on samples as published (19). Prior to biphasic extraction, an internal standard mixture consisting of 70 lipid standards across 17 subclasses is added to each sample (AB Sciex 5040156, Avanti 330827, Avanti 330830, Avanti 330828, Avanti 791642). Following two successive extractions, pooled organic layers are dried down in a Thermo SpeedVac SPD300DDA using ramp setting 4 at 35°C for 45 min with a total run time of 90 min. Lipid samples are resuspended in 1:1 methanol/dichloromethane with 10mM Ammonium Acetate and transferred to robovials (Thermo Fisher 10800107) for analysis. Samples are analyzed on the Sciex Lipidyzer Platform with an expanded targeted acquisition list consisting of 1450 lipid species across 17 subclasses. Differential Mobility Device on Lipidyzer was tuned with EquiSPLASH LIPIDOMIX (Avanti 330731). Instrument settings, tuning settings, and MRM list available upon request. Data analysis performed on an in-house data analysis platform comparable to the Lipidyzer Workflow Manager. Data analysis performed on an in-house data analysis platform comparable to the Lipidyzer Workflow Manager. Quantitative values were normalized to mg of tissue.

**SUPPLEMENTARY FIGURE LEGENDS**

**Supplementary Figure 1. Gfral deletion has no large effect on the degree of liver injury induced by Drp1 knockdown (NAG-Drp1si).**

**A)** ALT and AST level in wildtype or Gfral KO mice after 4 weeks of weekly injections with PBS or NAG-Drp1 siRNA (Drp1si). **B)** End-point plasma Fgf21 and Gdf15 protein content. **C-D)** Liver fibrosis and inflammation related genes *Col1a1, Col3a1, Acta2, Tgfb1, Timp1, Ccl2, Cd68, Adgre1* mRNA content were analyzed by qPCR. All data presented are the mean ± SD. n=8 *p<0.05, ****p<0.0001 one-way ANOVA.

**Supplementary Figure 2. Hepatocyte-specific Drp1 knockdown (NAG-Drp1si) induces mitochondrial oxidative damage, without causing changes in the expression of other mitochondrial dynamics genes.**

**A-C)** Lean male C57BL/6NTac mice were injected with PBS (control, n=8) or NAG-Drp siRNA (Drp1si, n=8) weekly for 4 weeks. All data presented are the mean ± SD. *p<0.05, ***p<0.001 two‐sided Student's t‐test.

**A)** Immunoblot of liver extracts measuring total content of dimeric and monomeric Prdx3, a mitochondrial antioxidant enzyme that dimerizes when ROS increase, after control or NAG-Drp1si treatments in non-reducing conditions. Total Prdx3 content was analyzed under reducing conditions. Gapdh was used as a loading control.

**B)** Densitometric analysis of Prdx3 of Western blots performed as in panel A. The ratio of dimeric and monomeric Prdx3 was calculated as surrogate to measure oxidative damage.

**C)** Liver Mfn1, Mfn2, Opa1 and Oma1 mRNA level analyzed by qPCR.

**Supplementary Figure 3: NAG-Drp1si treatment exacerbates the decrease in HOMA IR values induced by GAN diet feeding, changes the expression of mitochondrial dynamics genes and upregulates the expression of most Protein Phosphatase 1c subunits.**

**A-D)** C57BL/6NTac mice fed regular chow (RC) or GAN diet for 24 weeks before being injected weekly with PBS (control) or NAG-Drp siRNA (Drp1si) for 12 weeks. All data presented are the mean ± SD. RC/Control n=8, GAN/control n=12, GAN/Drp1si n=12, *p<0.05, one-way ANOVA

**A)** Fasting serum insulin.

**B)** HOMA-IR (insulin resistance) calculated by insulin (microIU/L) * plasma glucose (mmol/L) /14.1.

**C)** Liver Mfn1, Mfn2, Opa1 and Oma1 mRNA content quantified by qPCR.

**D)** Ppp1ca, Ppp1cb and Ppp1cc mRNA content quantified by qPCR.
