# Supplementary figures and images for: The mitochondrial fission protein Drp1 in liver is required to mitigate NASH and prevents the activation of the mitochondrial ISR
Source: Mol Metab. 2022 Aug 6;64:101566. doi: 10.1016/j.molmet.2022.101566 (PMC9420962; doi:10.1016/j.molmet.2022.101566)

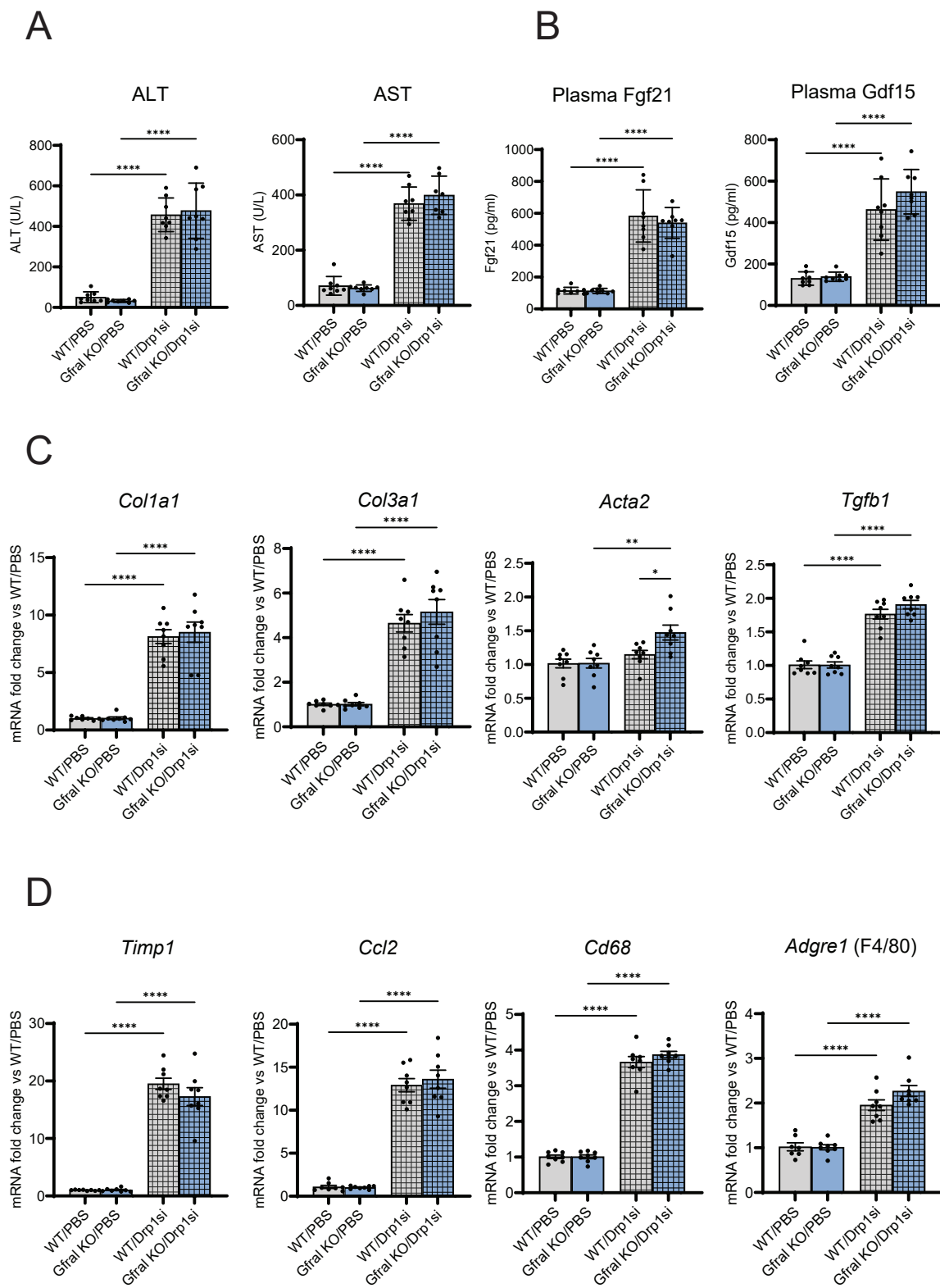

Supplementary Figure 1

A

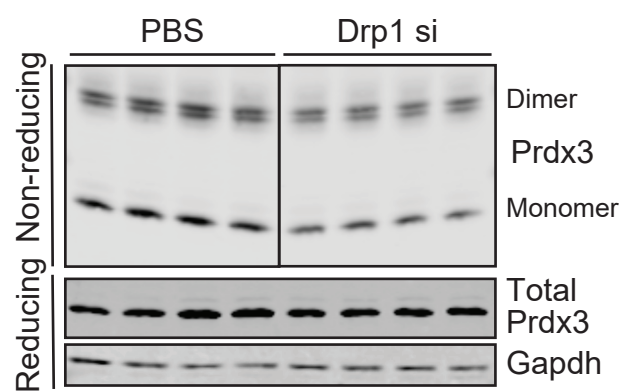

B

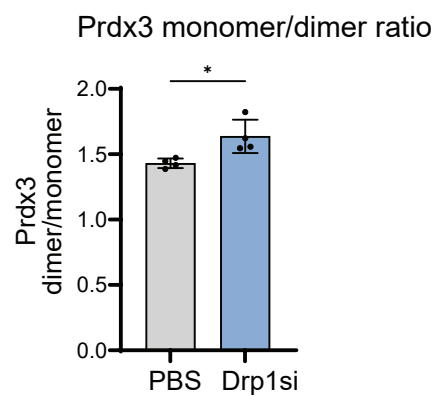

C

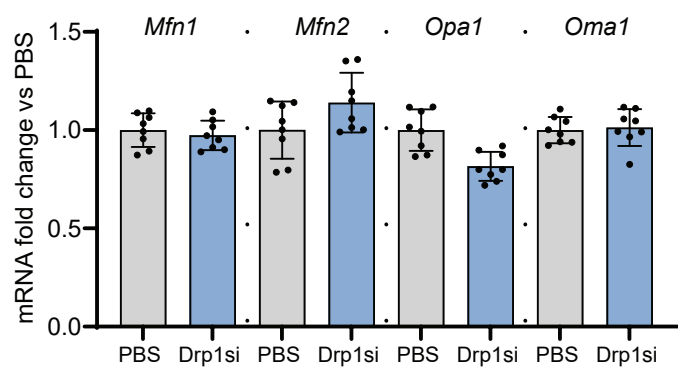

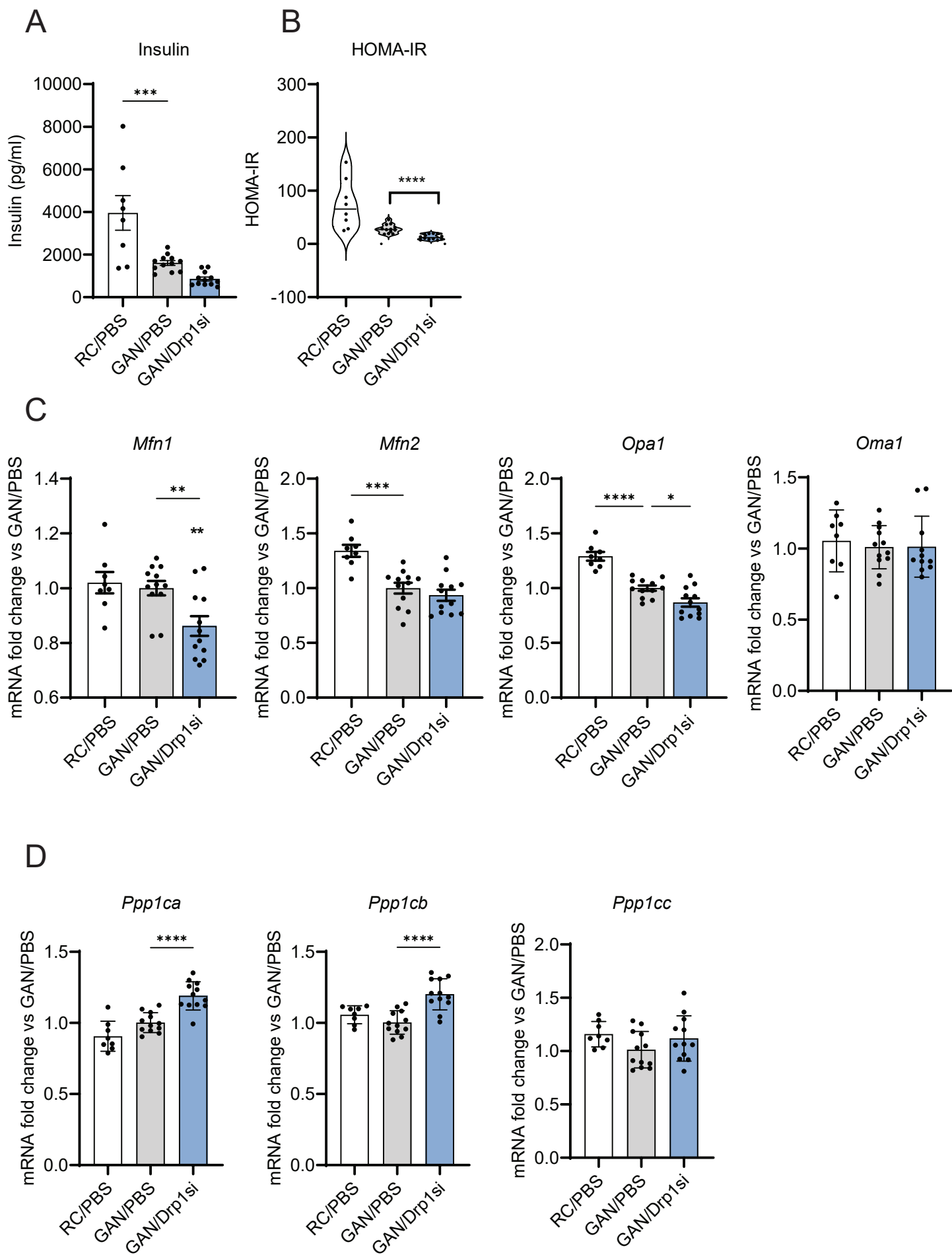

**Supplementary Figure 3**

Supplement: Multimedia component 2 [file mmc2.pdf]
